# Living in Mosul during the time of ISIS and the military liberation: results from a 40-cluster household survey

**DOI:** 10.1186/s13031-018-0167-8

**Published:** 2018-08-01

**Authors:** R. Lafta, V. Cetorelli, G. Burnham

**Affiliations:** 1 Department of Community Medicine, Al Mustansiriya University, Baghdad, Iraq; 2 Demographic and Social Statistics Section, United Nations Economic and Social Commission for Western Asia, Beirut, Lebanon; 3 Department of International Health, The Johns Hopkins Bloomberg School of Public Health, 615 N Wolfe Street, Baltimore, MD 21205 USA

**Keywords:** Mosul, Iraq, War, ISIS, Early marriage

## Abstract

**Background:**

In June 2014, an estimated 1500 fighters of the Islamic State of Iraq and Syria (ISIS) seized control of Mosul, Iraq’s second city. Although many residents fled, others stayed behind, enduring the restrictive civil and social policies of ISIS. In December 2016, the military activity, known as the liberation campaign, began in east Mosul, concluding in west Mosul in June 2017.

**Methods:**

To assess life in Mosul under ISIS, and the consequences of the military campaign to retake Mosul we conducted a 40 cluster-30 household survey in Mosul, starting in March 2017. All households included were present in Mosul throughout the entire time of ISIS control and military action.

**Results:**

In June 2014, 915 of 1139 school-age children (80.3%) had been in school, but only 28 (2.2%) attended at least some school after ISIS seized control. This represented a decision of families. Injuries to women resulting from intimate partner violence were reported in 415 (34.5%) households. In the surveyed households, 819 marriages had occurred; 688 (84.0%) among women. Of these women, 89 (12.9%) were aged 15 years and less, and 253 (49.7%) were aged under 18 at the time of marriage. With Mosul economically damaged by ISIS control and physically during the Iraqi military action, there was little employment at the time of the survey, and few persons were bringing cash into households. The liberation of Mosul in 2017 caused extensive damage to dwellings. Overall only a quarter of dwellings had not sustained some damage. In west Mosul, only 21.7% of houses had little or no damage from the conflict, with 98 (21.7%) households reporting their house had been destroyed, forcing its occupants to move. No houses had regular electricity and there was limited piped water. Inadequate fuel for cooking was reported by 996 (82.9%) households.

**Conclusion:**

The physical, and social damage occurring during ISIS occupation of Mosul and during the subsequent military action (liberation) was substantial and its impact is unlikely to be erased soon.

## Background

An estimated 1500 fighters of the Islamic State of Iraq and Syria (ISIS) seized Mosul in June 2014. While many people fled from Mosul, others entered from towns to the north of the city [1, 2]. The population remaining in Mosul under ISIS control was thought to be about 1.5 million [3]. A caliphate was established with a repressive bureaucracy to manage city affairs and control the life of its citizens [4, 5]. The Hesba morality police were created to ruthlessly enforce ISIS edicts.

Many public employees, including teachers and health workers, continued working under ISIS, while other persons were hired to replace persons who fled. Pay for these positions steadily deteriorated, yet people were expected to show up for work [6]. The Iraq Central government continued paying salaries of some civil servants into early 2015, though ISIS reportedly garnished 20–30% of these payments [7]. Payment of government pensions continued throughout the time. ISIS looted the Mosul banks, crushed enterprises and forcefully collected money from business owners and farmers to finance military campaigns. Many businesses collapsed and unemployment was widespread. Factories were dismantled and machinery sold in neighboring countries [2]. ISIS derived additional income sources from the sale of antiquities, increases in taxation and various financial penalties.

Educational curricula, from primary school to university, were rewritten to support ISIS’ radical views [8, 9]. Young women were at risk of forced marriages to ISIS fighters. At the university of Mosul, subjects such as law, arts and philosophy were dropped. Women could not study engineering and the sciences. Students in some technical courses and at university began to drift away, as they saw no future.

The military campaign to drive ISIS from Mosul, known as the liberaton, began on October 17, 2016. The attack on towns to the east of Mosul hearlded the beginning of the largest urban conflict since WWII [10]. Iraqi forces entered east Mosul on November 1, and declared it liberated on January 24, 2017. The second part of the campaign began with the attack on west Mosul on February 19, 2017. Military progress was slow, particulary in the old city, with widescale damage from artillary, IRAMs (Improvised Rocket Assisted Munitions) and airstrikes which obliterated whole neighborhoods. Fighting in west Mosul was concluded on June 29, 2017.

To assess living circumstances during the ISIS occupation of Mosul and the impact of the military liberation of Mosul on households, this study was undertaken when hostilities ceased.

## Methods

**Fig. 1:**
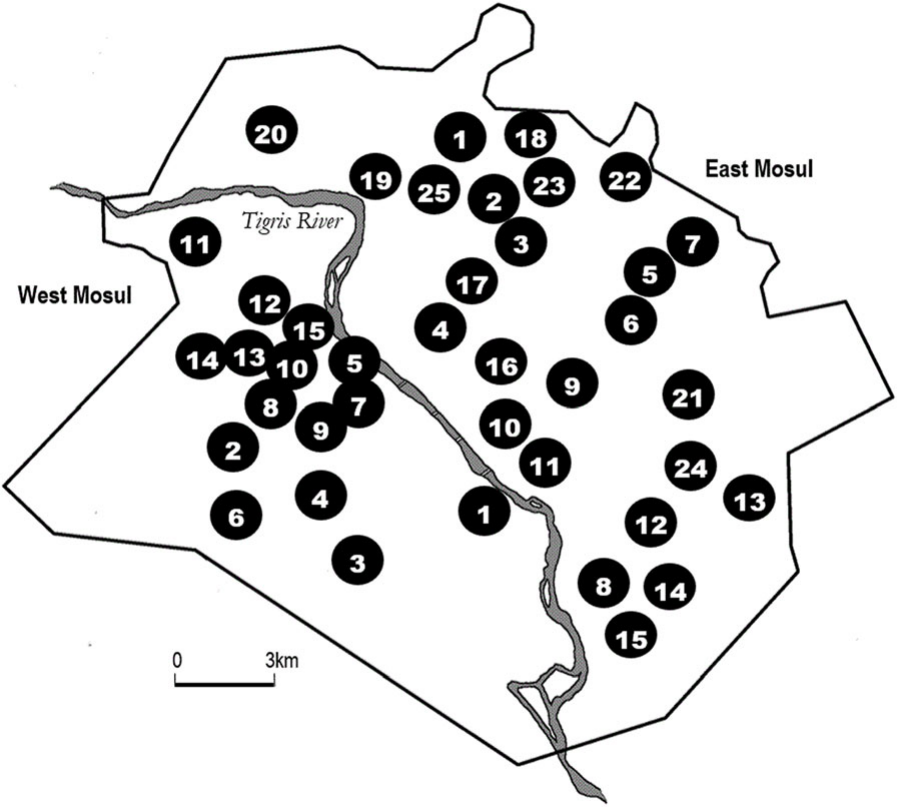
Location of clusters sampled in Mosul

**Fig. 2:**
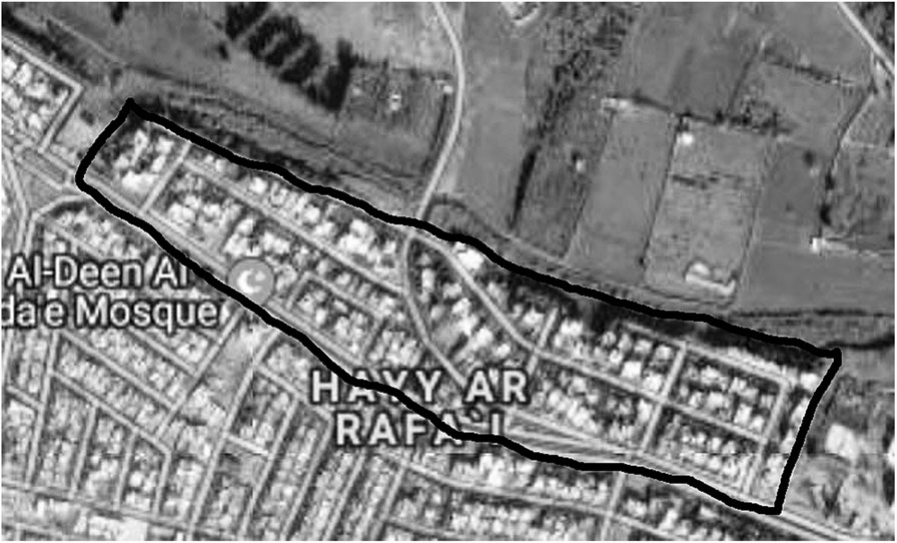
Satellite image of neighborhood 15, Hay Al Najar. Map data @2018 Google Imagery

This survey was conducted as soon as security would allow after the major military activity against ISIS had ceased in Mosul. Data were collected through a two-stage 40 cluster survey, with 30 households per cluster. Clusters were located randomly in selected administrative units, designated here as “neighborhoods” (Fig. 1). Based on the pre-ISIS population distribution of Mosul, 15 neighborhoods on the west side of the Tigris were chosen and 25 on the east side. There were no data available on population distribution in Mosul during ISIS occupation. Each neighborhood contained about 200–400 dwellings. A satellite image of survey site 15 is shown in Fig. 2. For a selected neighborhood, a 10 m grid was overlaid on a satellite image. A random location was selected. This was marked on the map which was transmitted to the study team along with the coordinates for this location. The survey team identified this location on the ground and selected the nearest dwelling as the start house. From the start house the teams moved to the nearest dwelling to the right until data from 30 households had been collected. When an intersection was reached, the teams always crossed to the right to continue until 30 household has been interviewed. For the survey, a household was defined as a group of people eating out of a common kitchen and in a dwelling with a separate entrance from the street, a definition which has proved satisfactory in previous Iraq household surveys [11]. The number of destroyed or unoccupied households in each health cluster was recorded. The survey questionnaire was based on those used in previous Iraq household surveys but adapted for Mosul circumstances. Adaptation was carried out with the assistance of Mosul health workers and faculty at Al Mustansiriya University. The questionnaire contained questions on living conditions, health needs, injuries and deaths. The final format was agreed among all the authors. The sample size of 1200 households was chosen as a size manageable by the number of qualified interviewers available, and utilizing experience from previous Iraq surveys, would provide sufficient details about household indicators. Results concerning health seeking behavior, injuries and deaths are being reported elsewhere.

Interviews were carried out by two teams, each with two interviewers who were Mosul physicians with doctorates in community medicine. Three of the four interviewers were female. Training was carried out for two days in Baghdad, and the questionnaire field tested in a non-survey area of Mosul. Only households which had been continuously present in Mosul since June 2014 were included. Verbal consent for the household to participate was obtained from the head of household. The informant was the senior female in the household, as the person most knowledgeable about household issues. No attempt was made to identify former ISIS supporters. Household interviews required about one hour and were conducted in privacy. Plans were made for interviewer protection including safe houses of their relatives and maintaining continuing cell phone communications.

The 25 clusters in east Mosul were surveyed between March 13 and 31, 2017. The prolonged fighting delayed the survey in west Mosul. Once access to west Mosul was gained, a new sampling frame was created to exclude the destroyed and depopulated neighborhoods of west Mosul. The 15 clusters of west Mosul were surveyed between July 18 and 31, 2017.

Five reserve neighborhoods were randomly chosen each for east and for west Mosul, should the original sites not be accessible. None were needed for east Mosul. For west Mosul two were randomly selected as replacements; one because of insecurity and the second to replace a neighborhood which was largely unoccupied.

The data were computer entered in Baghdad, with analysis carried out in Baghdad and Baltimore. Statistical analysis used Stata version 15 (Stata Corp, College Station, TX).

The study received ethical approval from the scientific and technical committee of Al Mustansiriya University, Baghdad. The analysis of deidentified data was exempted by the Institutional Review Board at Johns Hopkins Bloomberg School of Public Health as not human subjects research.

## Results

### Demographic information

Data from 1202 households were collected; 751 in east Mosul, and 451 in west Mosul. This represented a total of 7559 persons, 4867 living in east Mosul and 2692 in west Mosul. There were 2635 children under the age of 15 and 1643 women of child bearing age (Table 1). The average household size was 6.5 persons in east Mosul and 6.0 in west Mosul. No household declined to participate.

**Table 1:** Demographic characteristics of surveyed households

	East Mosul		West Mosul		All Mosul	
	No.	%	No.	%	No.	%
Young children (0–4 years)	632	13.0	353	13.1	985	13.0
Older children (5–14 years)	1071	22.0	579	21.5	1650	21.8
Male adults (15–49 years)	1172	24.1	650	24.1	1822	24.1
Female adults (15–49 years)	1126	23.1	517	19.2	1643	21.7
Older adults (50+ years)	866	17.8	593	22.0	1459	19.3
Total	4867	100.0	2692	100.0	7559	100.0

### Education

Education level of the household members (Table 2) listed only 167 (2.7%) Mosul household members with no formal education***.*** There were 2089 (33.6%) who were of school age (6 and older), and ordinarily would have been expected to have been in school. Among residents in the survey, 2670 (42.9%) had completed secondary school or above. In the 1202 households there were 915 (80.3%) children in school in June 2014. Following the ISIS take over, only 28 children (2.2%) had attended school at least at some point, but the majority of families decided to keep school aged children at home.

**Table 2:** Education levels of household members and school attendance of children

	East Mosul		West Mosul		Total	
	No.	%	No.	%	No.	%
Education level of household members^a^						
Adult with none	97	2.0	70	2.6	167	2.2
Under age for school^a^	964	17.8	472	17.5	1339	17.7
School age^a^	1383	28.4	706	26.2	2089	27.6
Primary school	911	18.7	383	14.2	1294	17.1
Secondary school	1020	21.0	736	27.3	1756	23.2
Post-secondary school	589	12.1	325	12.1	914	12.1
Education of children^a^						
In school in up to June 2014?						
Yes	304	67.4	611	88.8	915	80.3
No	147	32.6	77	11.2	224	19.7
Attended school after June 2014?						
Yes	3	0.5	6	2.0	9	0.1
No	591	96.4	300	98.0	891	97.0
Some	19	3.1	–	–	19	2.1
Reason not in school?						
School closed	4	0.7	–	–	–	–
Family decided	593	96.7	300	99.3	893	98.0
Taught at home	5	0.8	2	0.7	7	0.7
Left Mosul	10	1.6	–	–	10	1.1
Other	1	0.2	–	–	1	0.1

^a^school age in Iraq is 6 years

### Household relationships

**Fig. 3:**
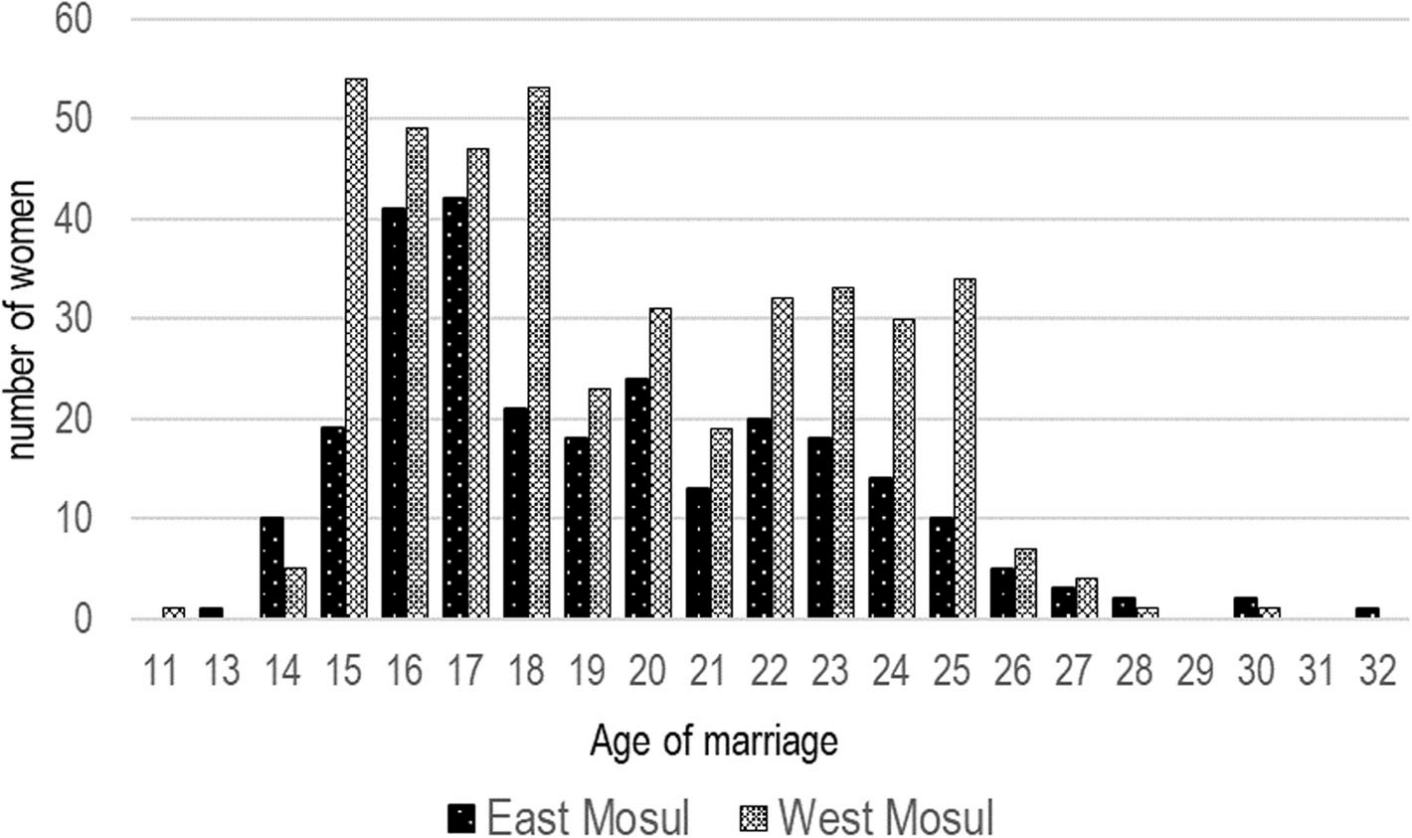
Age of Marriage for women in east and west Mosul 2014–2017

Since June 2014, there were 819 household members who were reported to have married (Table 3). Of these, 688 (84.0%) were females. The age of marriage was 15 years of less for 89 (12.9%) and 18 years and less for 253 (49.7%). The ages of marriage for females is shown in Fig. 3.

**Table 3:** Marriages since 2014, and intimate partner violence reported

	East Mosul		West Mosul		Total	
	No.	%	No.	%	No.	%
Sex of the person getting married						
Males	71	14.3	60	18.5	131	16.0
Females	424	85.7	264	81.5	688	84.0
Age of the person getting married						
10–19	232	46.9	155	47.8	387	47.3
20–29	254	51.3	156	48.1	410	50.1
30–39	9	1.8	12	3.7	21	2.6
40–49	–	–	–	–	–	–
50–59	–	–	1	0.3	1	0.1
Has there been any physical violence between husband and wife which led to injury to the wife since June 2014?						
Yes	132	17.6	283	62.8	415	34.5
No	612	81.5	168	37.3	780	64.9
Don’t know	7	0.9	–	–	7	0.6
Has there been any violence between someone in the household and someone from outside the household which has produced an injury since June 2014?						
Yes	80	10.7	5	1.1	85	7.1
No	665	88.6	444	98.5	1109	92.3
Don’t know	6	0.8	2	0.4	8	0.7

When violence at the household level was queried, there were reports of 415 injuries to women resulting from intimate partner violence, shown in Table 3. A further 85 persons had been injured from someone outside of the household, but not from direct military action. Questions about gender-based violence attacks occurring outside the household were consistently asked by interviewers, and respondents claimed they knew of none.

### Employment

When the informant was asked about employment status, the major categories were 353 (29.4%) with no employment and 442 (36.8%) indicating some part time work (Table 4). For those who said they were employed, this may have reflected a nominal employment status, but perhaps not one that was currently paying wages. When asked who had brought cash into the household in the past 2 months, there were only 304 persons identified from the 1202 households. In 61.2% of cases it was the wives who were bringing cash into the households.

**Table 4:** Livelihoods at the time of the interviews

	East Mosul		West Mosul		Total	
	No.	%	No.	%	No.	%
Current employment status of head of household^a^						
Clerical or office work	6	0.8	–	–	6	0.5
Manual labor, full time	70	9.3	4	0.9	74	6.2
Manual labor, part time	211	28.1	10	2.2	221	18.4
Market sales	131	17.4	–	–	131	10.9
Domestic work	23	3.1	–	–	23	1.9
Various intermittent casual work	222	29.6	–	–	222	18.4
No work	9	1.2	344	76.3	353	29.4
Retired	79	10.5	93	20.6	172	14.3
Persons earning cash during the past 2 months^b^						
Wife	122	62.9	64	66.0	186	61.2
Husband	55	28.4	20	20.6	75	28.0
Son	15	7.7	6	6.2	21	7.6
Daughter	–	–	1	1.0	1	0.7
Grandmother	1	0.5	1	1.0	2	0.7
Grandfather	–	–	2	2.1	2	0.7
Sibling of household head	1	0.5	3	3.1	4	1.3
Households with no cash income	4684	95.3	2595	96.3	7268	96.0

^a^ Nominal employment status even if not currently being paid

^b^11 households in east Mosul had 2 persons earning cash; in west Mosul there were no households with more than 1 person

### Structure and utilities

The assessment of household living circumstances at the end of the military activities show the difficult living circumstances being experienced (Table 5). Some 308 (41.0%) dwellings surveyed in east Mosul had no damage; 105 (14.0%) had major damage or were destroyed—forcing households to move. By contrast, in west Mosul only 2 dwellings (0.4%) were reported to be without some damage. There were 255 (56.5%) households which reported major damage to their dwelling, while 98 (21.7%.) reported their dwelling had been destroyed and they were forced to move. Kerosene and wood were major sources of energy, but wood was the major source of household energy for 310 (59.0%) households in west Mosul. Only 135 (11.2%) households reported they had adequate fuel for cooking. Electricity in Mosul was intermittently available to 656 (54.6%) households, with no households having a regular supply. In west Mosul, 389 (86.3%) reported no electricity. Water supply was obtained from wells for 1161 (96.6%) households. With carried water, 99.9% of flush toilets could be used.

**Table 5:** Living circumstances

	East Mosul		West Mosul		Total	
	No.	%	No.	%	No.	%
Damage to house in conflict						
None	308	41.0	2	0.4	310	25.8
Minor damage	338	45.0	96	21.3	434	36.1
Major damage	77	10.3	255	56.5	332	27.6
Destroyed and HH moved	28	3.7	98	21.7	126	10.5
Major source of energy						
Kerosene	439	58.5	183	40.6	622	51.7
Gas	2	0.3	1	0.2	3	0.2
Electricity	–	–	–	–	–	–
Wood	310	41.3	266	59.0	576	47.9
Other	–	–	1	0.2	1	0.1
Adequate fuel for cooking						
Yes	129	17.2	6	1.3	135	11.2
No	553	73.6	443	98.2	996	82.9
Sometimes	69	9.2	2	0.4	71	5.9
Availability of electricity						
Have almost all the time	–	–	–	–	–	–
Equal amount on and off	2	0.3	1	0.2	3	0.3
Mostly off	595	79.2	61	13.5	656	54.6
No electricity	154	20.5	389	86.3	543	45.2
Principal source of water						
Piped water	–	–	19	4.2	19	1.6
Tanker	–	–	–	–	–	–
Well	751	100.0	410	90.9	1161	96.6
River	–	–	16	3.6	16	1.3
Other	–	–	6	1.3	6	0.5
Presence of a working toilet						
Yes	750	99.9	442	98.0	1192	99.2
No	1	0.1	9	2.0	10	0.8

## Discussion

The data from this study reflect the impact on households of the 31–36 months of ISIS control of east and west Mosul, respectively. The damage to lives, the social structure of the communities and the education of children during this period is substantial. The consequences of the fighting to liberate Mosul from ISIS has created further suffering among Mosul households through the damage and destructions of dwellings, further deterioration in utilities, and shortage of cooking fuel.

Because of the fear of indoctrination and radicalization, almost all families kept their children from school. Many feared their older sons would be taken to be fighters during regular ISIS raids on schools [12]. Some children were taught at home, but most were not. This is a greater loss of education years than was previously documented for children from families fleeing the fighting in Al Anbar in 2015 [13].

In September 2014, the ISIS education bureau revised the curriculum for primary, secondary and university education, adding many religious topics, physical fitness and weaponry training. In addition, it imposed primary school fees of 15,000 dinars ($US12.60) per month as the Mosul economy worsened. ISIS threatened parents who did not send their children to schools. Teachers, especially women, were threatened with beheading if they did not report for classes [14]. Yet, families did not comply. Children have been traumatized by witnessing scenes of violence and executions of family, relatives and friends. Many children have lost family members or their entire family [15]. Since liberation, schools have reopened with unpaid teachers volunteering, and with children returning in numbers to recommence their education [16]. Recovering lost years of school will be impossible. Dealing with the psychological trauma from the brutality experienced by children is a great challenge [17]. The absence of education and the emotional trauma of the ISIS years combine to potentially cause considerable difficulties for children as they return to school.

The fear of forced marriages of daughters to ISIS members, especially foreign fighters, drove some residents to flee Mosul with their families. Many of those families remaining arranged marriages among relatives or other families. After marriage, many couples moved from Mosul where this was possible. This type of “protective” strategy to protect a daughter’s honor is described in conflict situations [18]. There were isolated reports of executions in Mosul over refusals of forced marriages to fighters, though overall, forced marriages did not seem to occur to the extent the population had feared [19]. It nevertheless remained a powerful fear and encouraged families to expeditiously marry their daughters, perhaps not always wisely. For Iraq as a whole, Unicef data indicate that 4.6% of girls under age 15 were married and 24.3%. of girls under age 18 were married [20]. In our survey population, 12.9% of marriages of women were age 15 and less, and 49.7% of marriages were for women aged 18 and less. A decrease in age of first marriage among women has been well documented [21, 22]. The reasons for these early marriage during conflict are often a complex mixture of cultural and economic reasons and sometimes were associated with lack of educational and employment options [23]. As the ISIS forces consolidated control of Mosul, young adult males also fled, fearing that if they stayed they could have no social contact with women and would be at risk of ISIS forced recruitment if they remained in Mosul. Marriage was not a protective strategy that worked for adolescent males who were liable to ISIS conscription regardless of marital status.

There were 34.5% of respondents (women) who reported intimate partner violence (IPV) leading to injury, which seems high, even by the reported levels in the Middle East [24, 25]. High prevalence of IPV among refugees and conflict-affected populations have been well documented, and are perhaps related to the economic, psychological and financial stresses of their situation [26–28]. These were all stresses present in Mosul. It is likely that these stresses will continue during what will be a difficult and prolonged recovery of the city.

Extensive destruction of housing affected all parts of Mosul. In east Mosul, 14.0% of houses sustained severe damage, with 3.7% uninhabitable. Almost all of this happened during the military liberation campaign. Even after the largely destroyed neighborhoods in west Mosul were dropped from the sampling frame, there were still 78.2% of houses which had sustained major damage, with about a third where the damage was so great that the household had been forced to move. The heavy use of artillery, IRAMs and airstrikes were reportedly responsible for much of the damage. However, airstrikes caused a disproportionate share of the human and infrastructural losses, particularly in west Mosul [29]. Some social and commercial life has returned to Mosul, especially in the less damaged east part of the city. The costs of rebuilding the entire city to its former state are in the billions of dollars and will require many years [30].

Water had been intermittently available during the ISIS occupation, though water treatment had faltered within the first year of the ISIS occupation [2, 31]. During the fighting in late 2017, a pipeline was hit which cut water to 40% of the city’s population [32]. Hand dug wells provided almost all water to households at the time of this survey [33]. Although households reported functioning flush toilets, these were functioning with water carried by hand. Electricity supplies had been steadily deteriorating for some time, but at the time of the survey it was mostly off, making it not possible to operate fans and air coolers when the temperature reached 45 °C [2]. During ISIS control the major energy source was kerosene. During the military liberation campaign, wood because a major source of fuel, particularly in west Mosul. Insufficient fuel for cooking was reported for most households, particularly in west Mosul [34].

### Limitations

There are many limitations to a study such as this conducted immediately following a major urban conflict. The sampling frame was initially based on pre-ISIS population distribution living in discrete administrative units or neighborhoods. While these neighborhoods each had approximately the same number of households before the ISIS occupation, the household numbers may have changed during the events after June 2014, potentially creating a sampling bias. This may have reduced representativeness, particularly in west Mosul, where many destroyed neighborhoods necessitated a substantial revision to the sampling frame. Still, after revisions to the sampling frame, two clusters had to be replaced, one because of insecurity, and a second because almost all of the remaining inhabitants had left. Even in the neighborhoods surveyed, many houses in the selected clusters were destroyed or deserted, particularly in west Mosul, creating a substantial survivor bias. As extended families tend to live in adjacent households, bias from clustering is likely.

## Conclusions

Data in this paper reflect the substantial impact ISIS occupation had on the city followed by the consequences of military action to destroy ISIS, particularly affecting west Mosul. The early marriage and the high prevalence of IPV, damage to dwellings, and households surviving without water and electricity are some of the stresses experienced. High unemployment, especially among males, was a further stress. Several years of education which were lost for students from grade school through to university that are difficult to replace. The costs of reconstructing Mosul and its economy will be high. The repair to lives damaged and destroyed by ISIS and recovery of lost opportunities will never be possible.
